# Time to HIV testing of sexual contacts identified by HIV-positive index clients in Siaya County, Kenya

**DOI:** 10.1371/journal.pone.0238794

**Published:** 2020-09-08

**Authors:** Paul Wekesa, Jaquin Kataka, Kevin Owuor, Lennah Nyabiage, Fredrick Miruka, Stella Wanjohi, Samuel Omondi

**Affiliations:** 1 Centre for Health Solutions, Nairobi, Kenya; 2 Division of Global HIV & Tuberculosis, Centers for Disease Control and Prevention, Kisumu, Kenya; 3 County Department of Health, Ministry of Health, Siaya, Kenya; University of the Witwatersrand, SOUTH AFRICA

## Abstract

There are no studies on time to test since notification among identified sexual contacts of HIV-positive index clients using program data in Siaya County and Kenya. We sought to understand time to HIV testing by contact characteristics after identification to inform targeted testing interventions. We retrospectively analyzed data from adult (aged ≥18 years) sexual contacts identified by HIV-positive index clients from 117 health facilities in Siaya County (June 2017–August 2018). We used Chi-square tests to assess for differences in characteristics of contacts by HIV testing. We performed Cox proportional hazards analysis and time to HIV testing of contacts analysis including time-varying covariates (cluster-adjusted by facility) to assess characteristics (age, sex, and relationship to index client) associated with time to HIV-testing since notification. Sexual contacts not tested were right censored at last follow-up date. We calculated hazard ratios with 95% confidence intervals to evaluate characteristics associated with time to testing. Of the 6,845 contacts included in this analysis, 3,858 (56.4%) were men. Most were aged 25–34 years (3,209 [46.9%]). Median time to contact testing was 14.5 days (interquartile range, 2.5–62). On multivariable analysis, contacts aged 18–24 years (aHR, 1.32 [95% CI: 1.01–1.73], p = 0.040) and 25–34 years (aHR, 1.18 [95% CI: 1.01–1.39], p = 0.038) had shorter time to HIV testing than those aged 35–44 years. Married polygamous (aHR, 1.12 [95% CI: 1.01–1.25], p = 0.039) and single contacts (aHR, 1.17 [95% CI: 1.08–1.27], p <0.001) had shorter time to HIV testing than married monogamous contacts. Non-spouse sexual contacts had shorter time to HIV testing than spouses, (aHR, 1.23 [95% CI: 1.15–1.32], p <0.001). We recommend enhanced differentiated partner services targeting older adults, married monogamous, and spouse sexual contacts to facilitate early diagnosis, same day treatment, and prevention in Western Kenya and sub-Saharan Africa at large.

## Introduction

Targeted HIV case-finding interventions such as partner services [1, 2], also referred to as assisted partner services or contact tracing, focus on ensuring that sexual partners of people living with HIV (PLHIV) are notified of their exposure, are offered testing, and are engaged in care [3]. Notification could be done through a passive or assisted approach, with the latter implemented through provider, contract, or dual referral methods [4]. Through partner services, screening efforts are targeted within specific networks of PLHIV going beyond family or household contacts, which previously were the focus of testing efforts in sub-Saharan Africa [2, 5].

With a high proportion of HIV serodiscordance in sub-Saharan Africa, timely HIV testing facilitates diagnosis and early access to treatment and care for those with new HIV diagnoses, access to prevention services for HIV-negative individuals, and support for various HIV services for both HIV-positive and HIV-negative individuals [6–10]. Reaching sexual contacts of HIV positive index clients could help prevent HIV transmission in serodiscordant relationships, especially among contacts of individuals with new HIV diagnoses [11]. Findings from a recent observational study in San Diego, CA, reported acute or early HIV infection in over one-third of contacts tested through partner services [12]. This suggests the need for early access to HIV-testing services for sexual contacts of index clients.

Early access to HIV testing has been demonstrated as feasible and successful. A cluster randomized controlled trial in Kenya demonstrated that sexual contacts of index clients using immediate assisted partner services were five times more likely to test for HIV within 6 weeks than the passive notification control group [13]. Sexual contacts who were randomized to immediate partner services had a higher proportion tested and receiving an HIV diagnosis for the first time compared to passive referral [13]. Partner services are cost-effective and a safe approach to decrease HIV morbidity and mortality rates in Kenya [14, 15]. However, losses after notification could limit access to HIV testing for sexual contacts. Some studies show losses between identification of sexual contacts to acceptance of testing, though some studies suggest improved linkage to care among those identified through partner services [16–18].

Understanding the time it takes to test notified contacts can help assess the effectiveness of partner services, but few studies have evaluated time to testing for sexual contacts of patients with a new HIV diagnosis among sexual contacts of HIV positive index clients notified. Partner services were implemented in program settings at health facilities in Siaya County in 2017. Siaya County has a high prevalence of HIV reported as between 15.3% (0–64 years) and 21% (15–49 years) [19, 20]. We evaluated time to HIV testing among sexual contacts notified through routine program implementation.

## Materials and methods

### Study design and setting

This cross-sectional retrospective study included 117 health facilities located in six sub-counties (Alego Usonga [Population in 2019: 224343], Bondo [Population in 2019: 197883], Gem [Population in 2019: 179792], Rarieda [Population in 2019: 152570], Ugenya [Population in 2019: 134354], and Ugunja [Population in 2019: 104241]) within Siaya County [Total Population in 2019: 993183] in the former Nyanza Province in Kenya [21]. Majority of facilities, 107, were government owned while six were privately owned and four were owned by faith based organizations. The health facilities were supported to provide HIV treatment and prevention interventions by Centre for Health Solutions—Kenya (CHS) through funding and technical assistance from the US Centers for Disease Control and Prevention (CDC) in partnership with the Ministry of Health and the Siaya County Department of Health. HIV testing services were provided at outpatient and inpatient service delivery points as well as at comprehensive care centers by HIV testing officers per Kenya’s HIV testing guidelines.

### Study population

The study population included adult (aged ≥18 years) sexual contacts identified by index clients with either a new or previous HIV diagnosis. Excluded from the analysis were adults living with HIV who did not identify sexual contacts or whose known sexual contacts had already undergone HIV testing. This analysis focused on the sexual contacts identified by index clients.

### Data collection

Routine data were collected by clinical and data officers using partner services registers (June 1, 2017–August 27, 2018). Variables included age, sex, marital status, relationship to index client (spouse or non-spouse), HIV test results, contact HIV test results, date contact was identified by the index client, date of contact’s HIV test, time to HIV test from the date of identification (if tested), and last follow-up at analysis dataset creation (August 27, 2018), facility and sub-county location. Spouse was used to mean the index client was married to the contact elicited while non spouse referred to contacts who were not married to the index from whom the elicitation was done. Data were entered into Epi Info Database, version 7.2 (CDC, Atlanta, GA), were exported into Microsoft Excel (Redmond, WA), and were imported for analysis into Stata version 15.1 (2017; StataCorp, College Station, TX).

### Data analysis

Counts, percentages, medians, interquartile ranges (IQR), and ranges were calculated. Chi-square tests were used to assess for differences in characteristics of contacts between those who underwent HIV testing and those who did not. Univariable Kaplan-Meier plots were used to determine HIV testing probability. Log-rank tests were used to determine differences in survival curves. Cox proportional hazards regression analysis was used to assess contact characteristics associated with a shorter time to test for HIV once identified by the index client. Contacts not tested by the data collection date (August 27, 2018) were right censored at their last follow-up date. The proportionality of hazards violation was checked using Schoenfeld residuals, scaled Schoenfeld residuals, and an overall global test. Any violation necessitated using a multivariable model in which time-varying covariates (TVC) interacted with the natural logarithm of time to HIV test to adjust for the violations over time. Significant TVC interaction indicated a modification of hazards over time. Sub-county variations were adjusted for in the multivariable models. All the regression models accounted for facility-level clustering. We calculated hazard ratios (HR) with their respective 95% confidence intervals (CI) and p-values. All statistical tests were evaluated at 5% significance level. All analyses were done using Stata version 15.1.

### Ethical approval

This study was approved by AMREF Ethics and Scientific Research Committee and was reviewed in accordance with the Centers for Disease Control and Prevention (CDC) human research protection procedures and was determined to be research. CDC investigators did not interact with human subjects or have access to identifiable data or specimens for research purposes.

## Results

### Sexual contacts’ characteristics and HIV testing

Of the 6,845 sexual contacts included in this analysis, 3,858 (56.4%) were men, and most (3,209 [46.9%]) were aged 25–34 years. Most sexual contacts (3,873 [56.6%]), were in married monogamous relationships. Slightly over half (3,781 [55.2%]) were non-spousal sexual contacts. Most were from Alego Usonga and Bondo sub-counties. Among the identified sexual contacts, 2,800 (40.9%) were tested for HIV; the rest were still being followed-up for HIV testing at the time of data analysis.

Of the 2800 contacts who were tested, a higher proportion of women (1,280 [45.7%]) were tested compared to those not tested (1,707 [42.2%]; p = 0.004). Significantly more sexual contacts in married monogamous relationships did not undergo testing (2,441 [60.3%]) compared to those who were tested, 1,432 (51.1%), whereas significantly more single contacts underwent testing (439 [15.7]) than those who did not (374 [9.2]; p<0.001). A significantly higher proportion of non-spouse sexual contacts were tested (1,832 [65.4%]) than those not tested (1,949 [48.2%]; p<0.001) as shown in Table 1.

**Table 1:** Characteristics of sexual contacts of HIV-positive index clients and HIV testing uptake in Siaya County, Kenya.

Sexual Contacts Characteristics	Total (n = 6,845)	Not tested (n = 4,045)	Tested (n = 2,800)	P value
	n (%)	n (%)	n (%)	
Sex				0.004
Female (n = 2,987)	2,987 (43.6)	1,707 (42.2)	1,280 (45.7)	
Male (n = 3,858)	3,858 (56.4)	2,338 (57.8)	1,520 (54.3)	
Age (years)				<0.001
18–24 (n = 1,085)	1,085 (15.9)	575 (14.2)	510 (18.2)	
25–34 (n = 3,209)	3,209 (46.9)	1,882 (46.5)	1,327 (47.4)	
35–44 (n = 1,691)	1,691 (24.7)	1,059 (26.2)	632 (22.6)	
≥45 (n = 860)	860 (12.6)	529 (13.1)	331 (11.8)	
Marital Status				<0.001
Married Monogamous (n = 3,873)	3,873 (56.6)	2,441 (60.3)	1,432 (51.1)	
Married Polygamous (n = 264)	264 (3.9)	170 (4.2)	94 (3.4)	
Single (n = 813)	813 (11.9)	374 (9.2)	439 (15.7)	
Unknown (n = 1,855)	1,855 (27.1)	1,037 (25.6)	818 (29.2)	
Widowed (n = 40)	40 (0.6)	23 (0.6)	17 (0.6)	
Relationship to Index Client				<0.001
Non-spouse Sexual Contact (n = 3,781)	3,781 (55.2)	1,949 (48.2)	1,832 (65.4)	
Spouse (n = 3,064)	3,064 (44.8)	2,096 (51.8)	968 (34.6)	
Sub-County				<0.001
Alego Usonga (n = 1,830)	1,830 (26.8)	1,034 (25.6)	796 (28.4)	
Bondo (n = 1,822)	1,822 (26.6)	1,065 (26.4)	757 (27.0)	
Gem (n = 1,078)	1,078 (15.8)	659 (16.3)	419 (15.0)	
Rarieda (n = 776)	776 (11.3)	403 (10.0)	373 (13.3)	
Ugenya (n = 616)	616 (9.0)	424 (10.5)	192 (6.9)	
Ugunja (n = 718)	718 (10.5)	455 (11.3)	263 (9.4)	

### Time to HIV testing and follow-up among identified sexual contacts

Median time to contact testing from notification of HIV exposure was 14.5 days (IQR, 2.5–62.0). Sexual contacts who were spouses and those in married monogamous relationships had the lowest median number of days to testing at 6.5 days (IQR, 0.5–35.5) and 7.5 days (IQR, 0.5–37.5), respectively. Men had a lower median time to test (14.5 days; IQR, 2.5–51.5) than women (16.5 days; IQR, 2.5–73.5). Among those not tested, the median follow-up duration was 173.5 days (IQR, 124.5–261.5). However, spouses not yet tested (194.5 days; IQR, 133.0–275.0) had longer follow-up durations than sexual contacts who were not spouses (159.5 days; IQR, 114.5–235.5) as shown in Table 2.

**Table 2:** Time to HIV testing and follow-up among identified sexual contacts of HIV-positive index clients in Siaya County, Kenya.

		Tested for HIV (n = 2,800)		Not Tested (n = 4,045)
Contact Characteristics		Median Days to HIV test (IQR)		Median Days on follow-up for HIV test for those not yet tested (IQR)
	n		n	
Overall Median	2,800	14.5 (2.5–62.0)	4,045	173.5 (124.5–261.5)
Contact Sex				
Female	1,280	16.5 (2.5–73.5)	1,707	175.5 (119.5–262.5)
Male	1,520	14.5 (2.5–51.5)	2,338	172.5 (125.5–259.5)
Contact Age, years				
18–24	510	18.5 (3.5–70.5)	575	186.5 (125.5–255.5)
25–34	1,327	13.5 (2.5–54.5)	1,882	175.5 (125.5–262.5)
35–44	632	15.5 (2.5–56.5)	1,059	168.5 (119.5–264.5)
≥45	331	16.5 (2.5–78.5)	529	165.5 (117.5–258.5)
Contact Marital Status				
Married Monogamous	1,432	7.5 (0.5–37.5)	2,441	185.5 (126.5–270.5)
Married Polygamous	94	13.5 (2.5–46.5)	170	182.5 (133.5–277.5)
Single	439	20.5 (5.5–66.5)	374	160.5 (116.5–252.5)
Unknown	818	35.5 (8.5–98.5)	1,037	161.5 (113.5–231.5)
Widowed	17	19.5 (5.5–56.5)	23	151.5 (111.5–236.5)
Relationship to Index Client				
Non-spouse Sexual Contact	1,832	21.5 (5.5–74.5)	1,949	159.5 (114.5–235.5)
Spouse	968	6.5 (0.5–35.5)	2,096	194.5 (133.0–275.0)
Sub-County				
Alego Usonga	796	9.5 (1.5–35.5)	1,034	199.5 (133.5–297.5)
Bondo	757	12.5 (1.5–51.5)	1,065	168.5 (125.5–257.5)
Gem	419	24.5 (4.5–96.5)	659	154.5 (108.5–238.5)
Rarieda	373	25.5 (3.5–68.5)	403	188.5 (119.5–264.5)
Ugenya	192	16.5 (2.5–79.0)	424	171.5 (112.5–222.5)
Ugunja	263	39.5 (8.5–104.5)	455	159.5 (117.5–237.5)

Abbreviations: IQR, interquartile range.

### Probability of HIV testing

**Fig 1:**
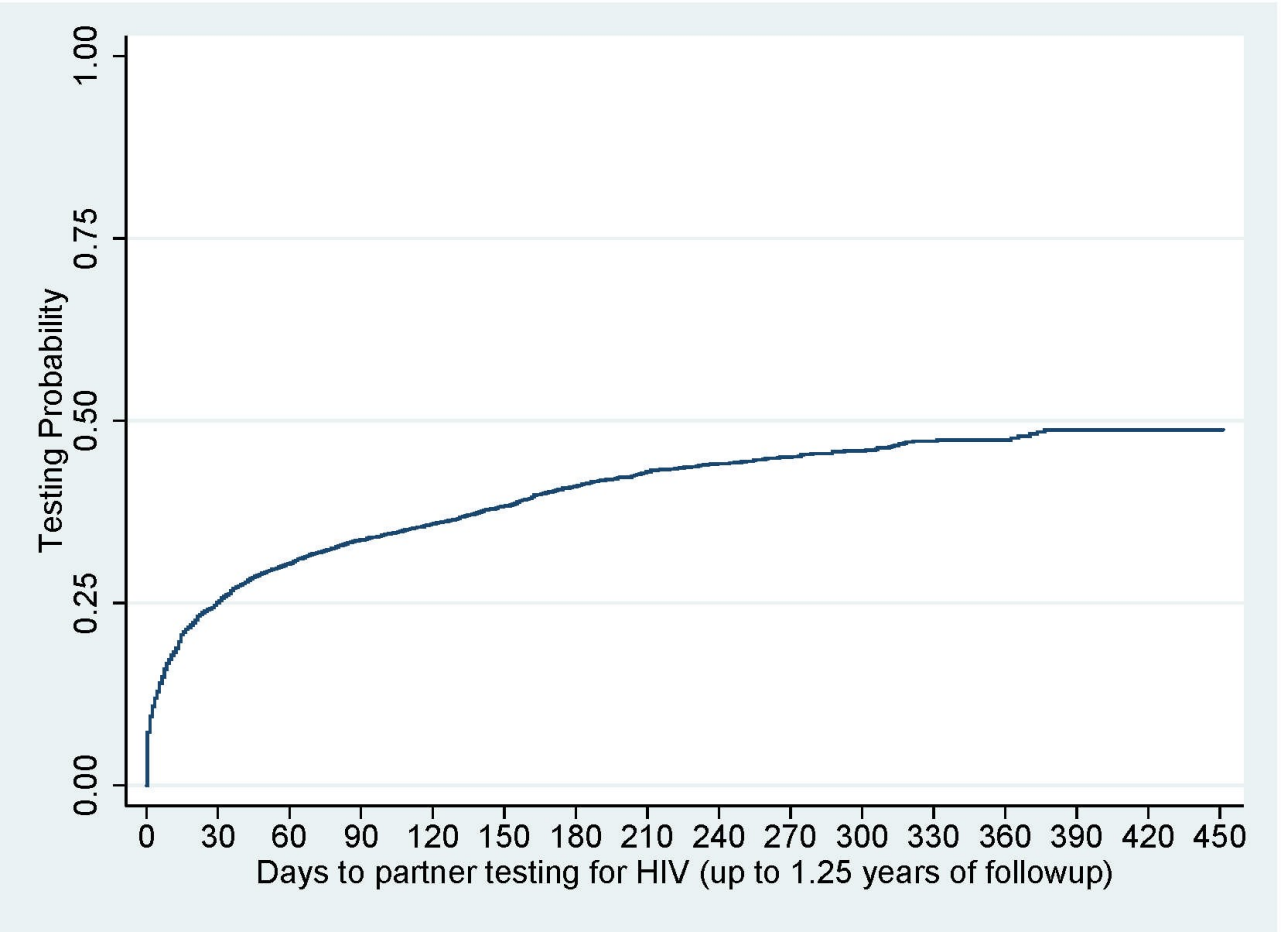
Overall probability of HIV testing among identified sexual contacts.

The overall probability of HIV testing among sexual contacts was 48.8% (95% CI: 46.8%–50.8%) over 1.25 years of follow-up (Fig 1).

**Fig 2:**
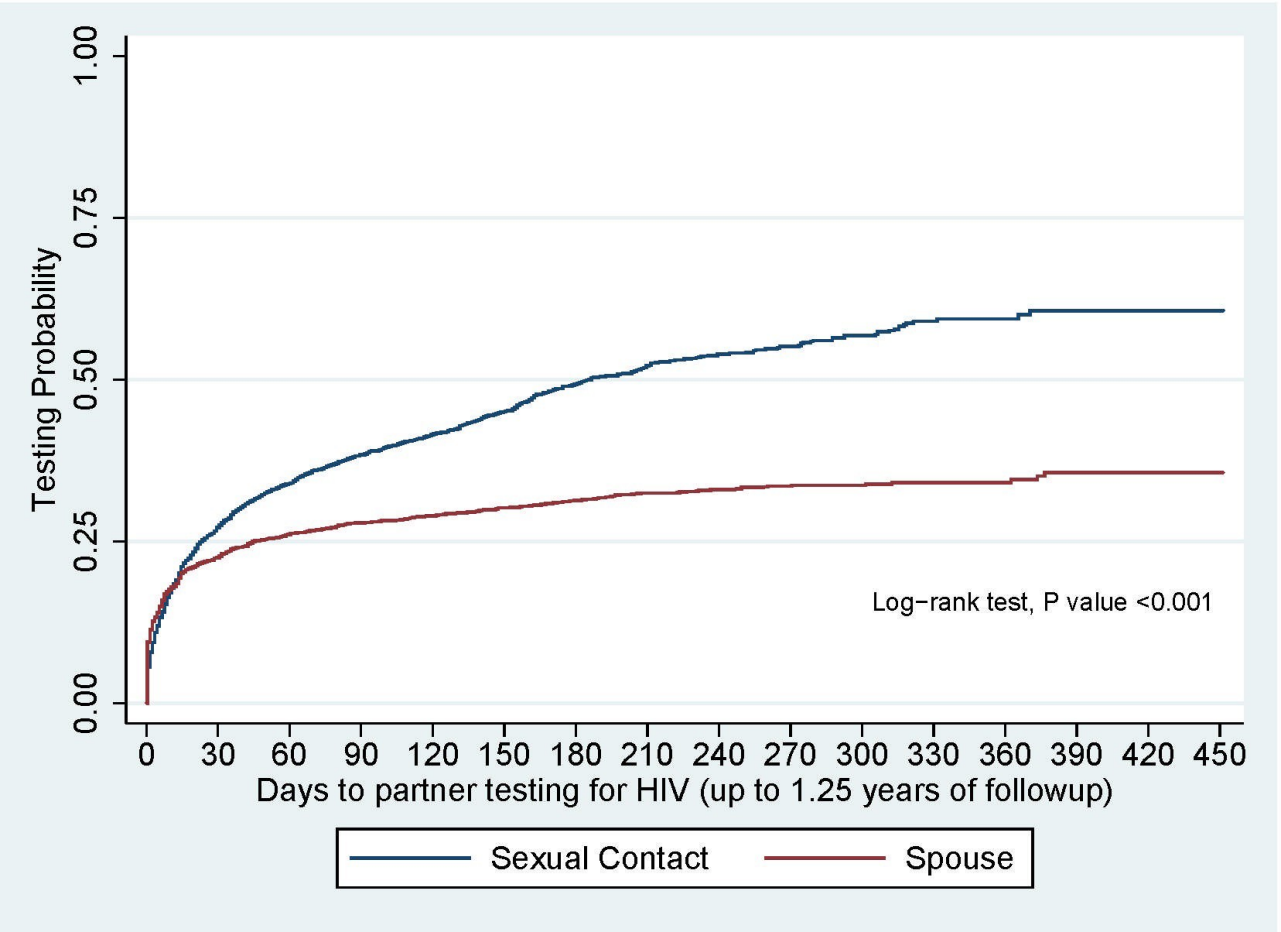
Contact time to test for HIV by relationship to index.

There was strong evidence to show that non-spouse sexual contacts had a significantly shorter time to test than spouses (p<0.001; Fig 2).

### Cox proportional hazards regression analysis of time to HIV testing

On univariable analysis, shorter time to testing was associated with younger sexual contacts aged 18–24 years (HR, 1.32 [95% CI: 1.15–1.51]) or 25–34 years (HR, 1.14 [95% CI: 1.04–1.24]), women (HR, 1.1 [95% CI: 1–1.12]), and single contacts (HR, 1.58 [95% CI: 1.25–2.0]). Shorter time to testing also was associated with being a non-spouse sexual contact (HR, 1.68 [95% CI: 1.44–1.96]) and living in Alego Usonga (HR, 1.49 [95% CI: 1.07–2.08]), Bondo (HR, 1.42 [95% CI: 1.02–1.97), or Rarieda sub-counties (HR, 1.65 [95% CI: 1.24–2.18).

On multivariable analysis, younger contacts aged 18–24 years (adjusted hazards ratio [aHR], 1.32 [95% CI: 1.01–1.73]) or 25–34 years (aHR, 1.18 [95% CI: 1.01–1.39]) were associated with shorter time to HIV testing. Age, contact marital status, relationship to index client, and sub-county violated the proportional hazards assumption. Results from the model including the TVC component indicated that married polygamous contacts (aHR, 1.12 [95% CI: 1.01–1.25]) and single contacts (aHR, 1.17 [95% CI: 1.08–1.27]) were associated with a shorter time to HIV testing compared to married monogamous contacts. Similarly, non-spouse sexual contacts were associated with a shorter time to HIV testing compared to spouses (aHR, 1.23 [95% CI: 1.15–1.32]; Table 3).

**Table 3:** Cox proportional hazards regression analysis of time to HIV testing among sexual contacts of HIV-positive index clients in Siaya County, Kenya.

Time to Testing	Univariable		Multivariable (Main Model)		Multivariable (Time-Varying Covariate)*	
	HR (95% CI)	P value	aHR (95% CI)	P value	aHR (95% CI)	P value
Age, years^§^						
18–24	1.32 (1.15–1.51)	<0.001	1.32 (1.01–1.73)	0.04	0.94 (0.87–1.02)	0.153
25–34	1.14 (1.04–1.24)	0.005	1.18 (1.01–1.39)	0.038	0.96 (0.9–1.01)	0.126
35–44	Ref		Ref		Ref	
≥45	1.03 (0.89–1.18)	0.727	0.96 (0.79–1.18)	0.727	1.02 (0.95–1.11)	0.54
Sex						
Female	1.10 (1–1.2)	0.04	0.95 (0.81–1.1)	0.496	-	-
Male	Ref		Ref			
Marital Status^§^^^^						
Married Monogamous	Ref		Ref		Ref	
Married Polygamous	0.93 (0.73–1.17)	0.527	0.78 (0.54–1.12)	0.174	1.12 (1.01–1.25)	0.039
Single	1.58 (1.25–2)	<0.001	0.71 (0.51–1)	0.05	1.17 (1.08–1.27)	<0.001
Unknown	1.18 (0.98–1.41)	0.079	0.43 (0.3–0.64)	<0.001	1.25 (1.14–1.38)	<0.001
Widowed	1.18 (0.77–1.81)	0.456	0.71 (0.29–1.74)	0.459	1.08 (0.82–1.42)	0.588
Relationship to Index Client^§^^^^						
Non-spouse Sexual Contact	1.68 (1.44–1.96)	<0.001	1.12 (0.84–1.48)	0.444	1.23 (1.15–1.32)	<0.001
Spouse	Ref		Ref		Ref	
Sub-County^§^						
Alego Usonga	1.49 (1.07–2.08)	0.019	1.90 (1.14–3.17)	0.014	0.90 (0.77–1.05)	0.186
Bondo	1.42 (1.02–1.97)	0.037	1.47 (0.92–2.35)	0.11	0.95 (0.82–1.09)	0.449
Gem	1.28 (0.98–1.67)	0.067	1.06 (0.73–1.54)	0.755	1.09 (0.93–1.28)	0.27
Rarieda	1.65 (1.24–2.18)	0.001	1.39 (0.9–2.15)	0.14	1.07 (0.91–1.25)	0.425
Ugenya	Ref		Ref		Ref	
Ugunja	1.15 (0.82–1.61)	0.417	0.71 (0.4–1.26)	0.242	1.20 (1.01–1.42)	0.038

*Time-varying covariate interacted with natural logarithm of time

^§^Violated proportional hazards assumptions and was included as a time-varying covariate in Cox model

^^^Significant time-varying covariate interaction

Abbreviations: HR, hazards ratio; CI, confidence interval; aHR, adjusted hazards ratio.

## Discussion

In our study, overall median time to testing of identified sexual contacts was 14.5 days, but other studies have presented varying time-to-test results. A cluster randomized controlled trial in Kenya reported a 67% testing rate of sexual partners within 6 weeks of enrolment of index clients. Those enrolled in the delayed group had only 13% testing in the period after 6 weeks [13]. Another randomized controlled trial in Malawi reported an overall return to clinic rate of 35%, and time to presentation to clinic of partners was associated with the notification approach. The 7-day median time to presentation was lowest (3 days) among locatable partners notified through passive referral [22]. Overall likelihood of testing in our study was 48.8% over 1.25 years is representative of this implementation setting but was higher than the Malawi trial and lower than the Kenya trial.

Sexual contacts who were married in polygamous relationships, who were single, or who were not married to the index client had a shorter time to testing for HIV. Other studies have suggested the importance of marital status in successfully referring contacts for HIV testing. A 2015 cross-sectional study in Tanzania demonstrated a higher rate of testing among sexual partners who were married [23]. Although similar to our findings, in our study, testing proportions were significantly higher among contacts in married polygamous relationships and among non-spouse contacts. The study in Tanzania showed a lower likelihood of referral and testing among sexual contacts who were casual partners or boyfriend/girlfriend compared to those who were married [23]. While this study does not report coverage, it was notable that a shorter time to test was found among non-spouse sexual contacts.

Younger sexual contacts had a shorter time to test than older contacts. A study evaluating data from 55 health departments in the U.S. suggested a higher likelihood of testing among younger sexual contacts aged 13–24 years compared with older partners aged 35–44 years [24]. Similarly, a cluster randomized controlled trial in Kenya reported a higher likelihood of younger sexual contacts opting for immediate as opposed to delayed assisted partner services [13]. While these studies did not evaluate time to test as an outcome, it was notable that while younger contacts had a shorter time to test in the model, the effect was not significant over time.

Early referral of sexual contacts is essential for access to HIV-testing services. Experiences in Malawi suggest that sexual contacts who are located and notified of their exposure were likely to seek testing services. Delays in notification decrease access to testing [25]. In our study, follow-up of identified sexual contacts could exceed 25 weeks, which delayed access to HIV prevention and treatment services and indicates the difficulty of reaching all sexual contacts in resource-constrained settings [25]. Notably, we found that non-spouse sexual contacts had shorter follow-up periods overall. In spite of these findings, the literature suggests that any service that encourages partner notification within implementation settings is beneficial in supporting access to HIV services [17].

Our study has several limitations. This study used routine implementation data; the available data and data collection tools were not specifically designed to investigate our research question and may have been incomplete and or have inaccuracies, which could bias our findings. The analysis methods used included robust multivariable regression methods adjusting for patient characteristics and facility level clustering to mitigate some of the bias. The study also was limited to health facilities within Siaya County, which limits the generalizability of our findings to other counties. The study included all identified sexual contacts eligible for HIV testing at CHS-supported health facilities, reducing participant-related biases. To the best of our knowledge, no other studies have investigated time to HIV testing among contacts identified through partner services in implementation settings in Kenya.

## Conclusions

Median time to HIV testing was about 2 weeks. Time to HIV testing was shorter among sexual contacts in married polygamous relationships, single sexual contacts, and among non-spouse sexual contacts. Overall, just under half of all sexual contacts were projected to access HIV testing over 1.25 years of follow-up. Understanding contact characteristics associated with time to HIV testing could help inform differentiated interventions aimed at improving testing service outcomes, to facilitate early diagnosis, same day treatment, and prevention in Western Kenya and sub-Saharan Africa at large.
